# Abnormal Large-Scale Neuronal Network in High Myopia

**DOI:** 10.3389/fnhum.2022.870350

**Published:** 2022-04-15

**Authors:** Yu Ji, Ling Shi, Qi Cheng, Wen-wen Fu, Pei-pei Zhong, Shui-qin Huang, Xiao-lin Chen, Xiao-rong Wu

**Affiliations:** Department of Ophthalmology, The First Affiliated Hospital of Nanchang University, Nanchang, China

**Keywords:** high myopia, resting state network, independent component analysis, functional connectivity, functional network connectivity

## Abstract

**Aim:**

Resting state functional magnetic resonance imaging (rs-fMRI) was used to analyze changes in functional connectivity (FC) within various brain networks and functional network connectivity (FNC) among various brain regions in patients with high myopia (HM).

**Methods:**

rs-fMRI was used to scan 82 patients with HM (HM group) and 59 healthy control volunteers (HC group) matched for age, sex, and education level. Fourteen resting state networks (RSNs) were extracted, of which 11 were positive. Then, the FCs and FNCs of RSNs in HM patients were examined by independent component analysis (ICA).

**Results:**

Compared with the HC group, FC in visual network 1 (VN1), dorsal attention network (DAN), auditory network 2 (AN2), visual network 3 (VN3), and sensorimotor network (SMN) significantly increased in the HM group. FC in default mode network 1 (DMN1) significantly decreased. Furthermore, some brain regions in default mode network 2 (DMN2), default mode network 3 (DMN3), auditory network 1 (AN1), executive control network (ECN), and significance network (SN) increased while others decreased. FNC analysis also showed that the network connection between the default mode network (DMN) and cerebellar network (CER) was enhanced in the HM group.

**Conclusion:**

Compared with HCs, HM patients showed neural activity dysfunction within and between specific brain networks, particularly in the DMN and CER. Thus, HM patients may have deficits in visual, cognitive, and motor balance functions.

## 1. Introduction

High myopia refers to myopia of −6 diopter or worse, a common eye disease worldwide (Morgan et al., 2012). By 2050, the estimated number of individuals with myopia or HM will reach 4.758 billion (49.8% of the total population) (Holden et al., 2016). In the past two decades, the incidence of HM has been increasing worldwide, particularly in Asia (Morgan et al., 2018). There are many reasons for HM, including genetic factors, environmental factors, and birth season (Wu et al., 2016); environmental factors can be divided into outdoor activities, close work, education, sex, age, and urban environment (Xiang and Zou, 2020). The typical pathological features of HM include diffuse or patchy chorioretinal atrophy, posterior staphyloma, varicella fissure, Fuchs spot, chorioretinal neovascularization, fovea (Ohno-Matsui et al., 2015), and ocular shape changes; the most important pathological changes are ocular shape changes, which can affect the optical power of the eye (Huang et al., 2018). Importantly, these pathological changes often lead to gradual decline in vision (Saw, 2006). In addition, the foveal avascular area increases in HM patients, while the vascular density decreases; these changes are closely related to fundus lesions (Min et al., 2020). HM can also induces various specific complications, including cataract formation, peripheral retinal tear-induced retinal detachment, myopic foveoschisis, macular hole with or without retinal detachment, peripapillary deformation, dome-shaped macula, choroidal/scleral thinning, myopic choroidal neovascularization, glaucoma, and potential blindness (Ikuno, 2017). Although the current ophthalmic examination enables a clear diagnosis of HM and its complications, there have been few studies of whether myopia can lead to changes in brain activity. In addition, Zhang X. W. et al. (2020) reported that HM patients had different amplitude of low-frequency fluctuations and default mode networks (DMNs), implying that cognitive function is influenced by HM. However, the pathophysiological mechanism by which HM causes changes in cognitive function remains unclear.

In recent years, resting state functional magnetic resonance imaging (rs-fMRI) has been widely used to analyze changes in brain network function among HM patients. Functional magnetic resonance imaging (fMRI) uses blood oxygen level-dependent (BOLD) signals that measure blood oxygen levels in the brain, which are determined by the amounts of oxygenated and deoxygenated hemoglobin. By measuring the strengths of these signals, fMRI can reflect the excitation states of neurons in the brain (Azeez and Biswal, 2017). The main advantage of rs-fMRI is that the signal can be repeatedly captured in a short period of time because BOLD signals do not require an external tracer; they provide comparisons of changes in the concentrations of oxygenated and deoxygenated hemoglobin during functional hyperemia (Hyder and Rothman, 2012). Although rs-fMRI is a non-interventional technique, it can accurately and reliably locate specific cortical areas of brain activity in space and time; it can simultaneously perform repeated scans of the target and track signal changes in real time. Using this technology, Zhai et al. (2016) demonstrated that FC was significantly reduced between the ventral attention and frontoparietal control networks in HM patients. Nelles et al. (2010) demonstrated that frontoparietal network activation was greater in ametropic patients than in normal controls. In a previous study, we showed that the low-frequency fluctuation amplitude was significantly reduced in the bilateral inferior frontal gyrus of HM patients, implying that HM patients have language comprehension and attention control disorders (Huang et al., 2016). However, the above studies have not performed sufficiently comprehensive neuroimaging analyses of spontaneous neural activity and brain network function changes in HM patients.

Independent component analysis (ICA) is a mathematical tool that can separate a series of mutually independent source signals from an observed mixture of unknown source signals, thereby solving the blind source separation problem. Electroencephalogram signals meet most assumptions of ICA (Urrestarazu and Iriarte, 2005); thus, ICA is particularly suitable for analyses of changes in brain network function among HM patients. In recent years, ICA technology has been widely used in studies of neuropsychiatric diseases, including brain network research in patients with stroke, Parkinson’s disease, and other diseases (Zhu et al., 2014, 2021). To our knowledge, the present study represents the first use of ICA to study spontaneous brain activity in HM patients, then to explore the relationship of spontaneous brain activity with behavioral performance in those patients.

## 2. Participants and Methods

### 2.1. Participants

This study included 82 HM patients and 59 healthy controls (HCs) who underwent examinations from August 2021 to December 2021 in the Department of Ophthalmology at the First Affiliated Hospital of Nanchang University. All participants were matched for age, sex, and education level. Individuals with brain diseases were excluded on the basis of medical history and physical examination findings. All participants underwent examinations in the same setting, and provided written informed consent to be included in the study. All procedures were performed in accordance with the Declaration of Helsinki; ethical approval was obtained from the Medical Ethics Committee of the First Affiliated Hospital of Nanchang University (Jiangxi Province, China).

The inclusion criteria for HM patients were: binocular vision of −6 diopters or worse; corrected visual acuity better than 1.0; and the completion of MRI-related examinations, optical coherence tomography, ultrasonography, and other ophthalmic examinations. The exclusion criteria for HM patients were: binocular vision of better than −6 diopters; history of ocular trauma or ophthalmic surgery; and/or neurological disorders and cerebral infarction.

Healthy controls were randomly selected from Nanchang City on the basis of their age, sex, and education level. All HCs met the following requirements: no eye diseases; no major diseases, such as neurological disorders or cerebral infarction; uncorrected vision or visual acuity better than 1.0; and the completion of MRI-related examinations, optical coherence tomography, ultrasonography, and other ophthalmic examinations.

### 2.2. fMRI Data Acquisition

All MRI data were obtained using a 3-TeslaTrio magnetic resonance imaging scanner system (Trio Tim, Siemens Medical Systems, Erlangen, Germany). These data included conventional T2-weighted imaging (T2WI) and fluid-attenuated inversion recovery (FLAIR) imaging for diagnostic and radiological assessment, along with high-resolution T1-weighted imaging (T1WI) for cortical surface complexity analyses. In this study, three-dimensional high-resolution T1WI parameters were as follows: repetition time/echo time = 1900/2.26 ms; field of view = 215 × 230 mm; matrix = 240 × 256; and 176 sagittal slices with thickness of 1.0 mm. Turbo spin echo-imaging sequence for T2WI scans was performed using the following parameters: repetition time/echo time = 5100/117 ms; field of view = 240 × 240 mm; matrix = 416 × 416; number of excitations = 3; echo train length = 11; and 22 axial slices with thickness of 6.5 mm. FLAIR imaging was performed using the following parameters: repetition time/echo time/inversion time = 7000/79/2500 ms; 50 slices; 240 × 217 matrix; and 0.43 × 0.43 × 2 mm^3^ voxels.

### 2.3. fMRI Data Processing

Statistical parameter mapping (SPM 12)^1^ was performed using the brain imaging data processing and analysis toolbox (DPABI^2^). Raw data were preprocessed as follows. First, the initial 10 time points of fMRI data were deleted to maintain the signal balance. Second, images were converted from DICOM format to NIFTI format. Third, slice timing correction, head motion correction, and interference covariate removal were performed for the remaining data. During preconditioning, if the participant’s head movement showed x-axis, y-axis, or z-axis rotation of >2.5° or >2.5 mm, the participant was excluded. Fourth, a single three-dimensional Bravo image was registered to the fMRI mean data; T1-weighted images were analyzed using the exponential Lie Algebra (DARTEL) toolbox to improve the spatial accuracy of fMRI data standardization. Fifth, the standardized data were resected at a resolution of 3 × 3 × 3 mm within the Montreal Neurological Institute (MNI) space. Finally, an isotropic Gaussian kernel of 6 × 6 × 6 mm full width at half maximum was used for spatial smoothing to reduce spatial noise and errors caused by spatial standardization.

### 2.4. Independent Component Analysis

Using group ICA of fMRI toolbox [GIFT (Calhoun et al., 2001)] software^3^ (version 3.0b), ICA was conducted to convert the preprocessed data into independent components. The process was as follows. First, the software estimated 36 independent components and calculated the spatial correlation of BOLD signals by using the minimum description length criterion. Second, all data for each participant were simplified. The compressed data set of each participant was joined into a group; the aggregated data set was dimensionally reduced using principal component analysis. Third, the independent components of each participant were reverse-reconstructed by ICA at the group level. Fourth, components of the brain network were screened using the following criteria: the spatial coordinates of the peak value of the brain network were located in the gray matter of the brain; there were no obvious blood vessels in the distribution of the brain network; and the brain network signal was a low frequency signal (0.1–0.25 Hz). Seven RSNs were identified in this study: VN, AN, DMN, SN, DAN, SMN, and ECN.

### 2.5. Statistical Analysis

SPSS version 20.0 software (SPSS Inc., Chicago, IL, United States) was used for statistical analysis. The means and standard deviations of sex, age, and axial length were determined in the HM and HC groups.

## 3. Results

### 3.1. Demographics and Visual Measurements

This study included 89 HM patients (41 men and 48 women, mean age 26.23 ± 5.462 years) and 59 HCs (24 men and 35 women, mean age 25.78 ± 3.102 years). Demographic characteristics are shown in Table 1.

**TABLE 1:** Demographic and clinical characteristics of HM and HC groups.

Characteristic	HM	HC
Men/women	41/48	24/35
Age (years)	26.23 ± 5.462	25.78 ± 3.102
ALM (OD)	26.67 ± 0.874	23.90 ± 0.971
ALM (OS)	26.58 ± 0.985	23.74 ± 0.693

*Abbreviations: HM, high myopia; HC, healthy control; ALM, axial length; OD, oculus dexter; OS, oculus sinister.*

### 3.2. Spatial Pattern of Resting State Networks in Each Group

**FIGURE 1:**
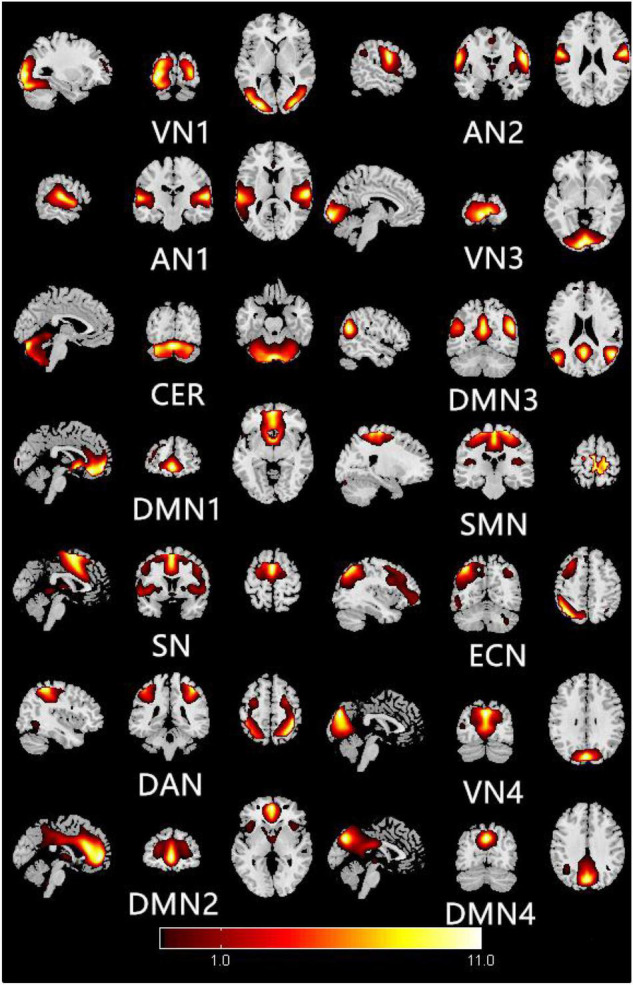
Typical spatial patterns of each RSN in HM group: visual network (VN), auditory network (AN), cerebellar network (CER), default mode network (DMN), sensorimotor network (SMN), saliency network (SN), executive control network (ECN), and dorsal attention network (DAN).

Figure 1 shows the typical spatial pattern in each RSN in the HM group. The visual network (VN) comprised the middle occipital gyrus, superior occipital gyrus, temporal-occipital regions, and fusiform gyrus; the auditory network (AN) comprised the bilateral middle and superior temporal gyrus. The cerebellar network (CER) comprised the cortex and medulla (parietal nucleus, intermediate nucleus, and dentate nucleus); the DMN comprised the posterior cingulate cortex, precuneus, medial prefrontal cortex, inferior parietal lobule, and lateral temporal cortex. The sensorimotor network (SMN) comprised the precentral gyrus, postcentral gyrus, and supplementary motor area; the salience network (SN) comprised the anterior insula and dorsal anterior cingulate cortex. The executive control network (ECN) comprised the dorsolateral frontal cortex and posterior cingulate cortex; the dorsal attention network (DAN) comprised the inferior parietal cortex, frontal eye motor area, auxiliary motor area, insular lobe, and dorsolateral prefrontal cortex.

### 3.3. Resting State Network Changes in the High Myopia Group

**FIGURE 2:**
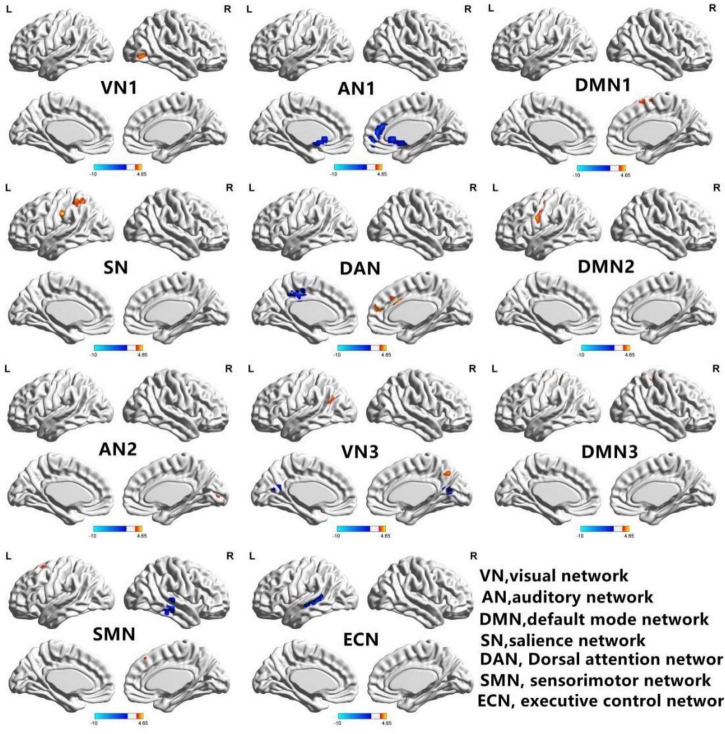
In the HM group, 11 brain regions of RSNs were significantly different (*P* < 0.01; GRF correction, cluster-level *P* < 0.05): visual network 1 (VN1), auditory network 1 (AN1), default mode network 1 (DMN1), significance network (SN), dorsal attention network (DAN), default mode network 2 (DMN2), auditory network 2 (AN2), visual network 3 (VN3), default mode network 3 (DMN3), sensorimotor network (SMN), and executive control network (ECN). BrainNet Viewer images show increased (warm colors) and decreased (cool colors) FC connectivities in the HM group.

Figure 2 shows the functional connectivity in each RSN in HM group. In VN1, the functional connectivity of the right middle occipital gyrus increased; in AN1, the functional connectivity of the left middle temporal gyrus decreased, while the functional connectivity of the left superior temporal gyrus increased. In DMN1, the functional connectivities of the left caudate nucleus and right anterior cingulate gyrus both decreased. In SN, the functional connectivity of the left middle occipital gyrus decreased, while the functional connectivity of the left insula increased. In DAN, the functional connectivity of the left parietal inferior gyrus region increased. In DMN2, the functional connectivities of the right medial superior frontal gyrus and right middle cingulate gyrus increased, while the functional connectivity of the left middle cingulate gyrus decreased. In AN2, the functional connectivity of the left central posterior gyrus region increased; in VN3, the functional connectivity of the right talar fissure region increased. In DMN3, the functional connectivity of the left precuneus region decreased, while the functional connectivities of the left angular gyrus and right precuneus region both increased. In SMN, the functional connectivities of the left posterior central gyrus and right anterior central gyrus both increased. In ECN, the functional connectivity of the right middle temporal gyrus decreased, while the functional connectivities of the left middle frontal gyrus, left inferior parietal gyrus, and left medial superior frontal gyrus all increased. The changes of RSN in HM group are shown in Table 2.

**TABLE 2:** Intranetwork FCs of RSNs in the HM group.

RSN	Brain region	BA	Peak t-score	MNI coordinates (x, y, z)	Cluster size (voxels)
VN1	R-MOG	19	4.6526	45, −75, 3	109
AN1	L-MTG	–	–4.4648	−42, −36, 0	64
	L-STG	42	4.1556	−63, −18, 9	108
DMN1	L-CAU	24	–4.4368	6, 21, −3	96
	R-ACC	–	–4.367	9, 42, 12	95
SN	L-INS	–	4.8092	−42, −18, 15	42
	L-MOG	–	–4.3905	−33, −81, 21	37
DAN	L-IPG	–	4.9254	−54, −21, 36	244
DMN2	R-SFGmed	–	5.5267	12, 48, 15	187
	R-MCC	32	5.2192	3, 21, 30	95
	L-MCC	–	–3.9378	−6, −24, 39	60
AN2	L-PoCG	–	4.6387	−36, −18, 45	186
VN3	R-CAL	17	3.3681	15, −81, 6	74
DMN3	L-PCNU	–	–4.1643	−6, −57, 18	52
	L-ANG	–	4.3362	−57, −54, 21	40
	R-PCNU	7	4.1531	6, −63, 42	59
SMN	L-PoCG	4	4.8034	−42, −15, 54	221
	R-PreCG	6	4.4296	24, −18, 57	244
ECN	R-MTG	21	–5.1214	66, −27, 0	47
	L-MFG	6	4.2445	−24, 12, 60	84
	L-IPG	–	3.4223	−45, −39, 48	32
	L-SFGmed	–	3.5085	0, 36, 45	41

*The statistical threshold was set at the voxel level with P < 0.01 for multiple comparisons using Gaussian random field theory (voxel-level P < 0.01, GRF correction, cluster-level P < 0.05). The t-score represents the statistical value of peak voxels showing differences in FC between the two groups. Abbreviations: MOG, middle occipital gyrus; MTG, middle temporal gyrus; STG, superior temporal gyrus; CAU, caudate nucleus; ACC, anterior cingulate and paracingulate gyri; INS, insula; IPG, inferior parietal; SFGmed, superior frontal gyrus, medial; MCC, median cingulate and paracingulate gyri; PoCG, postcentral gyrus; CAL, calcarine fissure and surrounding cortex; PCNU, precuneus; ANG, angular gyrus; PreCG, precentral gyrus; MFG, middle frontal gyrus; R, right; L, left; BA, brodmann area; MNI, montreal neurologic institute.*

### 3.4. Analysis of Functional Network Connections in the High Myopia Group

**FIGURE 3:**
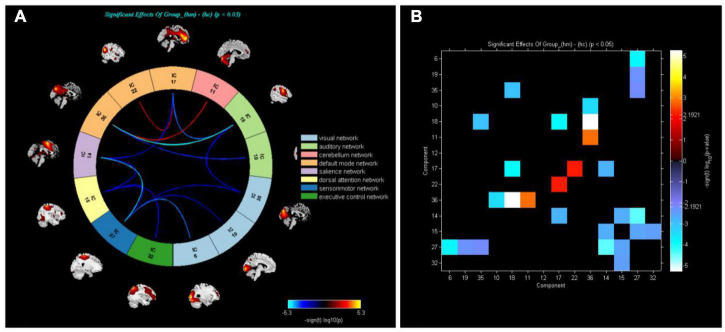
**(A)** Analysis of whole-brain functional network connectivity intensity allowed calculation of differences between HM and HC groups (*P* < 0.05, two-sample *t*-test). Yellow lines represent better functional connectivity than in HC group, while blue lines represent weaker functional connectivity than in HC group. **(B)** Group averaged static functional connectivity between independent component pairs was computed using overall resting state data. Values in the correlation matrix represent Fisher’s z-transformed Pearson correlation coefficients.

Figure 3 shows that functional connections of brain networks in HM group were analyzed by FNC technique. Network connections between DMN and CER were enhanced in the HM group (*P* < 0.05).

## 4. Discussion

To our knowledge, this is the first exploration of the changes and clinical characteristics of FC and FNC in the RSNs of HM patients. We found that, in HM patients, FC levels increased in VN1, AN2, DMN1, DMN2, DMN3, and SN; FC levels decreased in AN1 and ECN; and FC levels in DAN, VN3, and SMN both increased and decreased. Furthermore, the FNC of HM patients increased between DMN, AN, VN, SN, DAN, and SMN.

The VN is located in the occipital cortex at the temporal lobo-occipital junction, which has an important role in visual information processing (Wang et al., 2008). The main pathological changes in the VN of HM patients are excessive increase in ALM, the presence of posterior staphyloma, and multiple retinal and choroidal lesions (Liu et al., 2010; Ohno-Matsui et al., 2015). In addition, HM patients exhibit abnormalities in the visual cortex (Mirzajani et al., 2011). Zhang et al. (2021) reported that the response to spatial frequency visual stimuli was significantly reduced in HM patients; moreover, the resting state FC density between the superior limbic gyrus and the anterolateral prefrontal cortex was significantly reduced. Cheng et al. (2020) showed that FC density in the posterior cingulate cortex/anterior lobe region was significantly reduced in HM patients. Zou et al. (2021) demonstrated increased FC density between the VN and AN in patients with chronic migraine. Here, we found increased FC density in the right middle occipital gyrus and right talus fissure in HM patients. Taken together, the current and previous results show that long-term HM may lead to changes in FC density in the VN. Therefore, we speculate that HM patients have impaired visual function.

The AN is located in the temporal lobe and has an important role in auditory information processing. There is increasing clinical evidence that HM patients have impaired hearing function. Zhai et al. (2016) showed that FC density in the inferior temporal gyrus significantly decreased in HM patients. In addition, MRI analysis of chronic migraine patients showed that FC decreased in the AN, resulting in auditory disorders such as speech phobia (Moseley and Vlaeyen, 2015; Cordier and Diers, 2018). Zhou et al. (2019) demonstrated that FC density between the right inferior temporal gyrus and the right anterior central gyrus was enhanced in patients with tinnitus, while the right middle frontal gyrus showed a decrease in FC density between the bilateral cingulate gyrus and the left anterior central gyrus. Furthermore, we found showed that FC density in the left middle temporal gyrus of AN1 decreased in HM patients, while the FC density in the left superior temporal gyrus increased; additionally, FC density increased in the left postcentral gyrus region of AN2. Therefore, we speculate that HM patients have problems with auditory function. The specific underlying neural mechanism is unclear; we plan to investigate it in future studies.

The DMN consists of multiple brain regions, including the medial prefrontal cortex, posterior cingulate cortex, inferior parietal cortex, and anterior lobe (Raichle, 2015). The DMN has been linked to a wide range of neuropsychiatric disorders, including Alzheimer’s disease, autism, schizophrenia, and epilepsy. The DMN has an important role in self-referential thinking and reflection because it shows consistently high blood oxygen-dependent activity during rest (Raichle et al., 2001). There is increasing evidence that disorders of the central nervous system can lead to DMN abnormalities. Zuo et al. (2018) reported that when the brain shifts from a resting state to a low load state, FC density increases in the DMN; the VN also strengthens its interactions with other brain networks. Zhu et al. (2017b) showed that FC density in front of the DMN decreased in areca-dependent patients. Here, we found that FC density in the left caudate nucleus and the right angular gyrus of DMN1 decreased in HM patients; in DMN2, the FC density in the right medial superior frontal gyrus and the middle region of the right cingulate gyrus both increased, while the FC density in the middle region of the left cingulate gyrus decreased. Additionally, the FC density in the left angular gyrus and the right precuneus region of DMN3 increased, whereas the FC density in the left precuneus region decreased. Therefore, we speculate that HM patients have cognitive function impairment.

The SN, located in the dorsal anterior cingulate gyrus and anterior insula, guides behavior by participating in recognition of the most relevant stimuli (Menon and Uddin, 2010); it has an important role in the detection and location of explicit inputs and task control (Zhou et al., 2018). Zhang et al. (2019) demonstrated that the cortisol stress response level is associated with increasing FC density in the SN. The SN is also a key network for the detection of behavior-related stimuli, as well as the coordination of responses to such stimuli. Wang et al. (2016) reported that FC density in the SN decreases in schizophrenic patients, while the time of internal connection in the SN increases in a dynamic manner. Therefore, Sn dysfunction may be caused by the reduction of internal connection stability in the network. We found that FC density increased in the left insular region of the SN and decreased in the left middle occipital gyrus region of the SN in HM patients. Overall, the current and previous results indicate that long-term neurological disease may lead to changes in FC density in the SN. Therefore, we speculate that HM patients have impaired attention and working memory function.

The SMN is composed of subcortical areas such as the cerebellum, basal ganglia, and thalamus (Middleton and Strick, 2000), which can regulate bilateral body balance and control movement (Serrien et al., 2002). Liu et al. (2016) showed that movement disorders in stroke patients are related to abnormal FC density in the SMN or sensorimotor cortex. Acupuncture in stroke patients can increase FC density between the DMN and SMN (Cai et al., 2018). Zhang et al. (2018) reported that FC density in the SMN decreased in patients with lower limb amputation; FC changes in the brain revealed subcortical SMN abnormalities after amputation, highlighting the role of subcortical areas in SMN reorganization after lower limb amputation. In the present study, we found that FC density in the left postcentral gyrus and right precentral gyrus regions of the SMN increased in HM patients. Therefore, we speculate that HM patients have poor body balance and exhibit motor control disorders.

The DAN is composed of connected areas among the bilateral intraparietal sulcus, central anterior sulcus, and superior frontal sulcus (i.e., frontal eye movement area), which can provide top-down attention orientation. Thus, when the DAN is activated in daily life, it supports focused and efficient work. Xia et al. (2015) reported that FC density in the DAN of type 2 diabetes mellitus patients increased or decreased among regions, reflecting neurobehavioral defects in such patients. Wang et al. (2019) showed that FC density in the DAN decreased in patients with Alzheimer’s disease and mild cognitive impairment. Functional interactions between the DAN and DMN are dynamic at all stages of life; changes in FC density in the DAN may affect DMN function. Here, we found that FC density in the left inferior parietal gyrus of DAN increased in HM patients. Therefore, we speculate that HM patients exhibit impaired concentration and attention orientation.

The ECN is composed of the medial prefrontal cortex, cingulate gyrus, and posterior parietal cortex; these areas participate in activity inhibition and emotion, as well as multiple higher cognitive tasks, including adaptive cognitive control (Seeley et al., 2007). Zhu et al. (2018) demonstrated that FC density in the ECN decreased in patients who abstain from alcohol for an extended period; this FC density provided critical information for identifying alcoholism. Zhang Z. et al. (2020) showed that FC density in the right ECN decreased in patients with right frontal lobe epilepsy, while the FC density in the left ECN increased. These results suggest that repeated seizures and sustained epilepsy-related damage may lead to interaction disorders of core brain regions in the ECN, affecting network stability and consistency; these changes may lead to overall functional decline. Our study found that FC density in the left middle frontal gyrus, left inferior parietal gyrus, and left medial superior frontal gyrus of the ECN increased in HM patients, while the FC density in the right middle temporal gyrus decreased. Therefore, we speculate that HM patients experience obstacles to adaptive cognitive control.

Traditionally, HM is regarded as a visual impairment disorder; however, our FNC analysis showed that the network connection between the DMN and CER is enhanced in HM patients, indicating that visual impairment in HM patients may cause impairments in cognitive and balance functions. Zhang D. et al. (2020) showed that the network connection between the DMN and CER was weakened in patients with type 2 diabetes mellitus; this particularly affected the network connection between the bilateral cerebellar region IX and the right wedge/precuneus. The precuneus is the key hub of the DMN, with a vital role in emotional management (Sheline et al., 2009); the CER is also involved in emotional management. An abnormal connection between the left cerebellum and the posterior cortex/anterior lobe of the cingulate gyrus may be predictive of suicidal behavior in depressed adolescents (Zhang et al., 2016). Wang et al. (2017) studied the resting states of the cerebellar hemisphere and its subregions in 228 healthy adults; they found that network connections in various parts of the cerebellum were related to the DMN. Previous studies have shown that patients with alcoholism have weakened connections within the DMN, as well as between the DMN and other networks (including the CER) (Müller-Oehring et al., 2015; Zhu et al., 2017a). Wang et al. (2014) showed that visual loss can cause changes in high-level cognitive networks. Based on the above findings, we speculate that HM patients experience difficulty with cognitive and balance functions.

There were some limitations in this study. First, it constituted a small experimental analysis; we could not conclusively determine whether HM patients have non-visual dysfunctions (e.g., cognitive and balance functions); additional studies are needed to investigate these relationships. Second, although all participants underwent fMRI in the absence of any task requirement, the direct flow signals were presumably affected by physiological noise. Third, HM patients may have visual impairment complications (e.g., omental detachment, macular hole, and macular hemorrhage), which may cause changes in brain function that could influence the results of fMRI.

## 5. Conclusion

Through analyses of the FC within each brain network and the FNC between brain networks of HM patients and HCs, we found that HM patients may have defects in visual, cognitive, and motor balance functions, suggesting that abnormalities in FC may contribute to clinical symptoms in HM patients. Our results provide new insights concerning the pathophysiological mechanisms in HM. Furthermore, our report may support the use of rs-fMRI as a valuable, non-invasive examination of HM patients in regular clinical practice.

## Data Availability Statement

The raw data supporting the conclusions of this article will be made available by the authors, without undue reservation.

## Ethics Statement

The studies involving human participants were reviewed and approved by the Institutional Review Board of the First Affiliated Hospital of Nanchang University. Written informed consent to participate in this study was provided by the participants’ legal guardian/next of kin. Written informed consent was obtained from the individual(s) for the publication of any potentially identifiable images or data included in this article.

## Author Contributions

YJ, LS, and X-RW designed the study and oversaw all clinical aspects of study conduct and manuscript preparation. All authors contributed to data collection, statistical analyses, wrote the manuscript, designed the protocol, contributed to the MRI analysis, and approved the submitted version.

## Conflict of Interest

The authors declare that the research was conducted in the absence of any commercial or financial relationships that could be construed as a potential conflict of interest.

## Publisher’s Note

All claims expressed in this article are solely those of the authors and do not necessarily represent those of their affiliated organizations, or those of the publisher, the editors and the reviewers. Any product that may be evaluated in this article, or claim that may be made by its manufacturer, is not guaranteed or endorsed by the publisher.

## Footnotes

1
http://www.fil.ion.ucl.ac.uk

2
http://www.rfmri.org/dpabi

3
http://icatb.sourceforge.net/
